# COVID-19 Mortality in Children: A Referral Center Experience from Iran (Mofid Children's Hospital, Tehran, Iran)

**DOI:** 10.1155/2022/2737719

**Published:** 2022-03-16

**Authors:** Shahnaz Armin, Seyed Alireza Fahimzad, Sedigheh Rafiei Tabatabaei, Roxana Mansour Ghanaiee, Noushin Marhamati, Seyyedeh Narjes Ahmadizadeh, Azita Behzad, Seyedeh Masumeh Hashemi, Saeed Sadr, Maryam Rajabnejad, Mahnaz Jamee, Abdollah Karimi

**Affiliations:** ^1^Pediatric Infections Research Center, Research Institute for Children's Health, Shahid Beheshti University of Medical Sciences, Tehran, Iran; ^2^Pediatric Intensive Care Departmant, Mofid Children Hospital, Shahid Beheshti University of Medical Sciences, Tehran, Iran; ^3^Department of Pediatrics Pulmonary Diseases, Mofid Children Hospital, Shahid Beheshti University of Medical Sciences, Tehran, Iran; ^4^Pediatric Nephrology Research Center, Research Institute for Children's Health, Shahid Beheshti University of Medical Sciences, Tehran, Iran

## Abstract

**Background:**

The novel coronavirus disease 2019 (COVID-19) started in Wuhan, China, in December 2019. It spread widely around the world and was described as a pandemic by the World Health Organization (WHO). The knowledge regarding the mortality rate and risk factors of COVID-19 among the pediatric population is lacking. In this regard, we aimed to report the clinical and laboratory characteristics of deceased pediatric patients with SARS-CoV-2 infection.

**Method:**

This cross-sectional study was conducted in Mofid Children's Hospital, Tehran, Iran, from February 2020 to April 2021. Recorded documents of 59 pediatric patients (under 18 years old) assumed to have COVID-19 who had died in the COVID-19 ward and COVID-19 intensive care unit (ICU) were retrospectively evaluated. All statistical analyses were performed using SPSS software (v. 26.0, Chicago, IL). A *P* value of less than 0.05 was considered statistically significant.

**Results:**

From 711 COVID-19 definite and suspected patients, 59 children died. Of these deceased pediatric patients, 34 were boys (57.62%) and 25 were girls (42.37%), with a total mean age of 5.6 years. The median length of stay in the hospital was 10 days (range 1–215). 91.52% had underlying comorbidities of which neurological diseases accounted for the largest share. 54 patients were admitted to the ICU and 83.05% of them had intubation during their hospitalization. In addition, the most common reasons for death in our study were related to respiratory and multiorgan failure.

**Conclusion:**

According to our knowledge, we are the first team to report such a thorough study in the field of COVID-19 pediatric mortality in Iran. Mortality was observed in all age groups of children, especially in those with previous comorbidities, specifically neurological disease. Abnormally elevated tests of ESR, CRP, LDH, AST, and ALT as well as the presence of proteinuria and hematuria were found in more than 50% of patients in our investigations, and ICU admission between both definite and suspected groups had significant differences, so monitoring and considering these factors may help to control and reduce the progression of the disease to death.

## 1. Introduction

The novel coronavirus disease 2019 (COVID-19) caused by the severe acute respiratory syndrome coronavirus 2 (SARS-CoV-2) has received global attention since its first diagnosis in Wuhan, China, in December 2019. Due to the alarming levels of prevalence, severity, and stagnation, this outbreak was described by the World Health Organization as a pandemic [1] and has since emerged as a major public health emergency with global destructive effects [2]. This is the first pandemic caused by the coronavirus [3]. Based on the Lynx Insight Service report on April 2 (Reuters), more than 129.34 million people have been infected with the new coronavirus worldwide and 2,947,927 have died. Since the first cases were identified, infections have been reported in more than 210 countries and territories [4].

Nevertheless, the significant numbers of asymptomatic children (about 25%) [5] and the longer incubation period [6] compared with adults impose a potential threat of silent distribution of the infection. Most pediatric patients represent mild symptoms [7], and few studies worldwide have reported severe forms of COVID-19 in children [8–10]. Severe acute respiratory syndrome coronavirus 2 (SARS-CoV-2) infection is assumed to be rare in children, accounting for 1.7–2% of diagnosed patients [11]. In the pediatric population, the impact of comorbidities, such as chronic kidney diseases, neurological disorders, cardiovascular disease, liver disease, and inborn errors of immunity affecting the interferon pathway (as the most frequent disorders) [12–15] on the outcome of SARS-CoV-2 infection is disputed [16, 17]. Due to the paucity of studies published on pediatric COVID-19 infections [8], including critically ill patients, a better understanding of the mortality rate and risk factors associated with COVID-19 infections in pediatric patients is still lacking.

The objective of this study is to report the clinical and laboratory characteristics of deceased pediatric patients with SARS-CoV-2 infection.

## 2. Methods

From February 2020 to April 2021, 711 COVID-19-infected patients (477 definite and 234 suspected) were admitted to Mofid Children's Hospital, a referral center in Tehran (capital city of Iran), and we retrospectively evaluated 59 pediatric patients who died with the diagnosis of COVID-19 infection (definite or suspected).

We categorized patients into definite or suspected; cases that met two or more of these criteria were considered suspicious [18]:

- A high fever, malaise, or gastrointestinal or respiratory symptoms
- Laboratory data with a decrease in white blood cells (WBC) count or lymphocytopenia or an increase in C-reactive protein (CRP)/erythrocyte sedimentation rate (ESR)
- Atypical chest imaging, as well as computed tomography (CT) scans

And they are classified as definite if they meet the following criteria [18]:

- COVID-19 nucleic acid positivity in blood samples, nasal, and pharyngeal swab samples, as confirmed by RT-PCR

A questionnaire was developed and filled with demographic and laboratory data, length of hospital stay (LOS), underlying comorbidities, clinical manifestations, and outcomes.

The SPSS software (v. 26.0, Chicago, IL) was used for the statistical analyses. Descriptive statistics included means and standard deviation (SD) for normally distributed or medians and interquartile range (IQR) for skewed measures, and proportions for categorical variables. A *P* value <0.05 was considered statistically significant.

## 3. Results

This study was conducted between February 2020 and April 2021 on 59 COVID-19 patients (with definite or probable novel coronavirus infection) who died in Mofid Children's Hospital, Tehran, Iran. 34 were males (57.62%) and 25 were females (42.37%), with a total mean age of 5.6 years.  Table 1 shows the number of pediatric patients in each age group in total.

The median days of hospitalization were 10 (range 1–215).

About 89.83% of the study population had underlying medical conditions; 31 patients (52.54%) had only one and 22 (37.29%) had two or more underlying diseases.

In total, patients, in the course of their illness, experienced most of the following symptoms: 52 (88.1%) had respiratory distress, 50 (84.7%) dyspnea, 39 (66.1%) fever, and 27 (45.8%) cough. Also, 39 (66.1%) showed gastrointestinal (GI) manifestation, and 28 (47.5%) showed varying degrees of loss of consciousness in the period of illness.

The most common clinical symptoms seen in our patients are listed in Table 2.

In our study population, at the time of admission with diagnosis or suspicion of COVID-19, 47 (79.7%), patients had either no or mild dehydration and 12 (20.3%) had moderate or severe dehydration; among the latter group, 58.3% (7 patients) had the moderate type and 41.7% (5 patients) showed the severe type of dehydration.

54 patients (91.52% of the study population) were admitted to the ICU, and 49 (83.1%) of them had been intubated during their hospitalization. 51 (86.4%) of cases had chest CT in favor of COVID-19 during their illness.

Based on the mentioned criteria for definite and suspected identification patterns, we had 54 patients that could be considered in these two groups.

34 out of 54 (63%) were known as definite, based on a positive COVID-19 PCR test.

**Figure 1:**
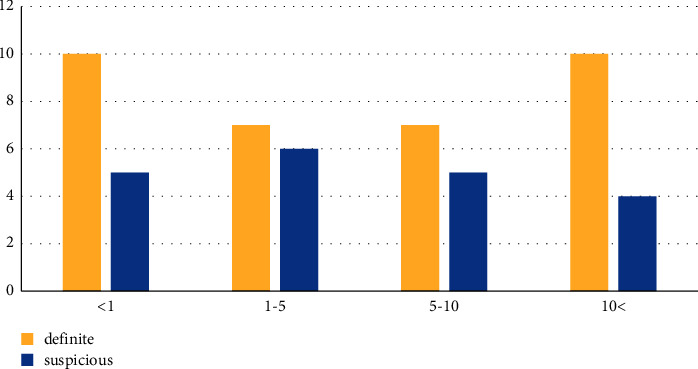
Age chart of the definite and suspicious groups.

Half of the definite patients (17/34) and 65% of the suspected cases (13/20) were males. In the definite group, the children's mean age was 6.36, and in the suspicious group, this mean number was 4.9 years (Figure 1).

The median length of stay (LOS) in definite children was 13.5 days, which was more than 1.5 times the probable one (8 days).

18 (90%) of suspected children and 32 (94.11%) of definite ones had different underlying diseases (Table 3).

In both groups, the mean range of some initial tests, including ESR, LDH, AST (aspartate aminotransferase), ALT (alanine transaminase), and CPK (creatine phosphokinase), was higher than normal.

**Figure 2:**
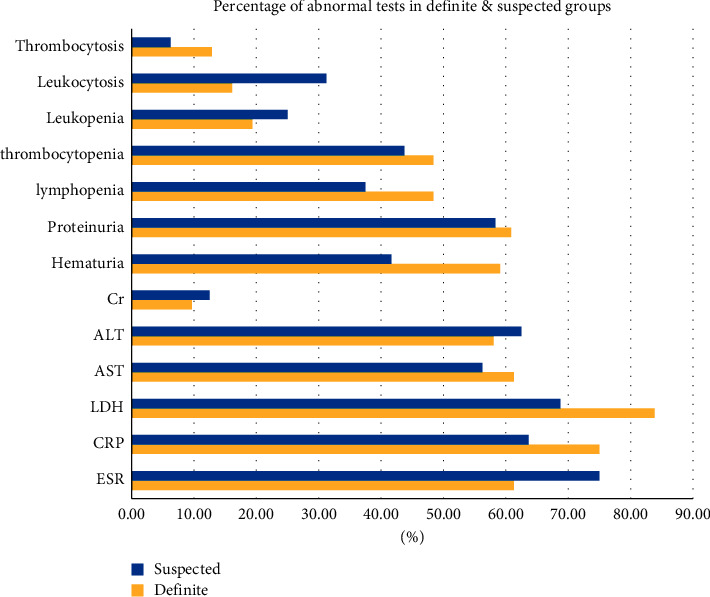
Percentage of abnormal initial tests in the definite and suspected groups.

In Figure 2, we compare the level of tests in the definitive and suspected groups. Statistical analysis shows there was no significant difference between these groups.

Also, according to the reference of this hospital, we considered albumin less than 3 abnormal, which after analysis and comparison of the two definite and suspicious groups, no significant difference was found (the mean range of initial albumin in these two groups was 3.51 and 3.14, respectively).

16 (80%) of the suspected group and 33 (97.06%) of the definite group had been admitted to the ICU during their hospitalization (with a *PP* value of 0.037). 15 (75%) of the first group and 29 (85.3%) of the second one experienced intubation through their confinement.

In the suspected group, 95% of CT scan findings were in favor of COVID-19 while this number was 87.1% in the definitive group.

Except for ICU admission (with a *P* value of 0.03), in general, no significant relationship was found in *P* value less than 0.05 between definite and suspicious groups in symptoms and laboratory tests.

The reasons for the death in our total group of pediatric patients are shown in Table 4.

## 4. Discussion

Since the worldwide studies on SARS-CoV-2 mortality in children are limited or information that has been collected is insufficient, according to our evidence, in the field of COVID-19 pediatric mortality in Iran, we are the first group to report such a study with this number of data and variables over a long time.

In the children's group, some recent epidemic study results, such as Dong et al.'s results, suggest that boys are dominant in getting affected by the virus; however, with a slight predominance of boys, there was no significant difference in getting infection between the sexes [10]. Also, in our study, boys were dominant with 57%.

There is a risk of infection and mortality in all age groups of children with COVID-19 infection [19]. From studies of Olivia et al. in the United Kingdom, Philip et al. in New York City, and the CDC, the severity and mortality of COVID-19 were higher in children under age 1 and adolescents [20–22]. In the same way, our study shows the highest number of deaths in the age group under one year, then 10 years and above, after that 1–5 years, and finally 5–10 years, respectively.

From reports at Pernambuco-Brazilian in 2020, on patients under 20 years, based on pediatric age group, the neonates and infants' fatality rate was higher [23]. Also, in reports from Jakarta, Indonesia, in a hospital-based retrospective cohort study, death happened across all age groups, and based on pediatric age-specific mortalities, the highest death percentage in children was for 0–4 years [24].

According to a study by Rutgers researchers, children, adolescents, and young people with underlying comorbidities are more at risk for SARS-CoV-2 severe complications [25]. A significant number of our total COVID-19 patients who died had the underlying disease (91.52%) was observed. In addition, the Aimen et al. pediatric study reported a 7% fatality rate in which they clearly stated the presence of underlying diseases is considerably associated with mortality [26]. In proportion to these statements, children with underlying diseases should be taken care of as much as possible [27].

In France, Oualha et al.'s study on severe and fatal forms of a novel coronavirus in pediatric cases announced that 70% of cases had comorbidities, most of which were neurological ones with 25.92% [8]. In Singh et al.'s cohort study of COVID-19 children mortality, about 11.11% had a neurological underlying illness [28], but in ours, about 33.33% had this condition. However, the presence of underlying cardiac, hematologic, and gastrointestinal diseases in suspected COVID-19 patients who died was higher than in the definitive group, so attention to this issue in the field of care is likely to be helpful. However, in some pediatric studies, such as two case series in China by Wang et al. and in Iran by Rahimzadeh et al., the underlying disease was not present among children diagnosed with COVID-19 [29, 30]. This difference may be due to the severity of the disease in the study population. Our hospital is an excellent multidisciplinary referral center for children, so cases with underlying diseases and COVID-19 infection may be referred here. In addition, in this study, only cases related to deceased children were investigated.

From 171 confirmed children, according to Lu et al. studies, 48.5% showed cough as the most common symptom [31]. Also, in another study, as stated by Wu et al., one-third of 74 COVID-19-infected children had cough and fever during their disease duration [32]. The most common symptoms in our observations were somehow similar to the results of other countries' studies [10, 20, 33–35].

According to the studies, which Harvard University also reported, under hydrated conditions, body temperature regulation, prevention of infection, delivery of nutrients to cells, and proper functioning of organs are established. In order to create balanced hydration and support the body's healthy metabolism, drinking an adequate amount of water continuously is essential. Dehydration is associated with a number of health disorders [36]. It is also expected that suboptimal hydration in the weeks prior to COVID-19 exposure increases the risk of mortality from this infection [37]. In a COVID-19 study from the United States, David and his colleagues reported that one of the most common symptoms for patients who died with MIS-C (after cough, nausea, dyspnea, and loss of appetite) was dehydration (6 of 14, 43%) [38], while in our study, out of 59 patients, 20.3% of children were in the category of moderate and severe dehydration.

From pooled analysis and review of Henry et al. in severe COVID-19 pediatric patients, out of 5 studies, 61.5% of cases had elevated CRP, and also, out of 3 studies, 72.7% presented elevated LDH [39]. We also experienced increased LDH and CRP levels, with a percentage of 78.72 and 71.43, respectively. The elevation of these markers indicates a serious infection or inflammation.

In the New Delhi cohort study, of fatal cases of confirmed SARS-CoV-2 infection in children, 44.4% of them had increased AST and ALT [28], which was similar to our data.

From reviewing several reports, Henry et al. found a normal leukocyte count in 69.2% of pediatric cases and only 3% of cases had lymphopenia [40]. Besides, the Lu et al. study states that lymphopenia was present in just 3.5% of COVID-19-infected children [31]. In another study by Wang et al., 30% of infants showed this abnormal situation [41]. On the contrary, in the Lippi and Plebani study of adult patients, the prevalence of increasing the number of leukocytes during disease progression was noted [42].

In our study, less than 45% of patients had lymphocytopenia and only a limited number of patients had thrombocytosis (10.64%). Leukocytosis also affected less than a quarter of the population. We should consider that our study was done on a poor prognostic group which may be shown differently from other children's COVID-19 case data.

In our study, a significant difference was observed between the two groups of definite and suspicious COVID-19 in the field of ICU admission, which seems reasonable. We noticed that endotracheal intubation was done in most cases, a procedure in which the likelihood of transmitting the virus and infection is very high [43, 44].

Based on our retrospective study in Mofid Children's Hospital, Tehran, Iran, the fatality rate over 14.5 months was 8.29%.

The mortality rate of this novel virus varies from country to country. In Norway, an archival study of over 2.5 months (1 March 2020–15 May 2020) did not report any confirmed SARS-CoV-2-related deaths in children under 20 years of age [45]. From Brazilian reports by Sena et al. in 2020, out of 682 confirmed pediatric cases in the state of Pernambuco, the total mortality rate was 5.6% [23]. According to the American Academy of Pediatrics (APP) and the Association of Children's Hospitals (CHA) in April 2021, in the United States, children diagnosed with COVID-19 account for 13.8% of the total. 1.2 to 3.1% of COVID-19 patients and 0.00–0.21% of COVID-19 deaths are pediatric [46]. Furthermore, patients under the age of 20 accounted for 2.1 percent with a mortality rate of 0.02 percent, according to an analysis of COVID-19 cases confirmed by the Centers for Disease Control and Prevention (CDC), China [47].

According to a study of COVID-19 deaths in children at a hospital in Indonesia, Dewi and her colleagues reported that two of the most common causes of death were acute respiratory distress syndrome and septic shock [48]. However, the priority of our findings as the causes of death was first respiratory diseases and then multiorgan failure which may be due to the high rates of underlying diseases in our study population.

## 5. Conclusion

According to our information in the field of COVID-19 pediatric mortality in Iran, this is the first original report about the death of children from COVID-19. As a result, children with comorbidities, especially neurological diseases, that are prone to suffer from serious illnesses should receive better care. In addition, children with fever, changes in the level of consciousness, and clinical, gastrointestinal, and respiratory symptoms need more attention. There was a significant difference in the rate of ICU admission between the definite and suspicious groups, and since the most important organs involved in coronavirus disease are the lungs, it seems logical that in our study, the most common causes of death are in the respiratory category.

## 6. Limitations

As in all other retrospective studies, data were collected from patients' medical files and there were some missing data or some variables did not request at all. We suggest a closer look for other pediatric studies on definite cases who died of COVID-19.

## Figures and Tables

**Table 1:** Age groups of patients in total children.

Total age range	Number of children	Percentage
<1	17	29
1–5	14	24
5–10	13	22
10<	15	25

**Table 2:** Common symptoms in total patients.

Symptoms	Percentage	Numbers in total
Respiratory distress	88.1	59
Dyspnea	84.7	59
Fever	66.1	59
Gastrointestinal	66.1	58
Loss of consciousness	47.5	59
Cough	45.8	58

**Table 3:** Types of underlying diseases in the definite/suspected groups.

Underlying disease	Definite (%)	Suspected (%)
Malignancy	23.53	10
PID^*∗*^	11.76	15
Neurologic	35.29	25
Hematologic	5.88	20
Rheumatologic	11.76	0
Respiratory system	8.82	10
Endocrine	11.76	25
Renal	14.71	15
Hepatic	8.82	10
Gastrointestinal	5.88	10
Traumatic	2.94	0
Cardiac	8.82	25

^*∗*^Primary immunodeficiency diseases.

**Table 4:** Reasons for death in COVID-19-infected children.

Reasons for death	Percentage	Numbers in total
Respiratory	54.2	59
Multiorgan failure	16.9	59
DIC	13.6	59
Septic shock	8.5	59
Sepsis	6.8	59
Shock	5.1	59
CNS	5.1	59
Renal	3.4	59
Hepatic	1.7	59
Cardiac	1.7	59
Hemodynamic	1.7	59

## Data Availability

The data used to support the findings of this study are included within the article.

## Abbreviations

MIS-C: Multisystem inflammatory syndrome in children
DIC: Disseminated intravascular coagulation
ESR: Erythrocyte sedimentation rate
CRP: C-reactive protein
LDH: Lactate dehydrogenase
AST: Aspartate aminotransferase
ALT: Alanine aminotransferase
RT-PCR: Real-time reverse-transcriptase polymerase chain reaction.
